# A Sequential Algorithm for Signal Segmentation

**DOI:** 10.3390/e20010055

**Published:** 2018-01-12

**Authors:** Paulo Hubert, Linilson Padovese, Julio Michael Stern

**Affiliations:** 1 Instituto de Matemática e Estatística, University of São Paulo (IME-USP), São Paulo 05508-090, Brazil; 2 Mechanical Engineering Department, Escola Politécnica—University of São Paulo (EP-USP), São Paulo 05508-010, Brazil

**Keywords:** signal detection, bayesian methods, hypothesis testing, audio segmentation

## Abstract

The problem of event detection in general noisy signals arises in many applications; usually, either a functional form of the event is available, or a previous annotated sample with instances of the event that can be used to train a classification algorithm. There are situations, however, where neither functional forms nor annotated samples are available; then, it is necessary to apply other strategies to separate and characterize events. In this work, we analyze 15-min samples of an acoustic signal, and are interested in separating sections, or segments, of the signal which are likely to contain significant events. For that, we apply a sequential algorithm with the only assumption that an event alters the energy of the signal. The algorithm is entirely based on Bayesian methods.

## 1. Introduction

Signal processing is a field of intense research, both in engineering, physics and medicine (for a quick and interesting exposition on the applications of signal processing, see https://signalprocessingsociety.org/our-story/signal-processing-101. For subacquatic signal processing, see [1]), and in the statistical (particularly Bayesian) literature [2,3,4]. The most recurrent problems in the field are signal estimation, model comparison, and signal detection [5,6,7]: in signal estimation, given a functional form for the signal (for instance, an exponentially decaying tonal model) and a sample, the researcher wants to estimate the functional form’s parameters (the rate of decay, the fundamental frequency). In model comparison, there are different possible functional forms for the signal, and given one or more samples, one is interested in selecting the most adequate model for them. In signal detection, the researcher is given a sample of the signal, and must decide if a given functional form (which we might call an event) is or is not present.

These situations arise typically when the researcher has some control over the process of data acquisition. Namely, she is usually well aware of the presence of the event of interest in the sample, or knows precisely the kind of event she is looking for (and/or its duration). In these situations, previous works from the 1980s and 1990s [3,5,6,7] have successfully applied maximum entropy and Bayesian methods to solve the basic problems of signal estimation, model comparison and signal detection.

On the other hand, there are situations in which the researcher has very little information about the occurrence times or precise functional form of events. This is the case in this work.

We analyze samples of a subaquatic audio recording obtained by the OceanPod, a hydrophone developed by the Laboratory of Acoustics and Environment (LACMAM) at EP-USP. The OceanPod is capable of storing 5 months of a digital signal sampled with a frequency of 11.025 Hz. More information about the OceanPod can be found at http://lacmam.poli.usp.br/Submarina.html.

In January 2015, one OceanPod was first installed in the region of Parque da Laje, a marine conservation park on the Brazilian coast, in the region of Santos, SP. It is kept at a depth of 20 m, and after a period ranging from 30 to 90 days it is retrieved for data extraction. After the data is extracted, the hydrophone is reinserted at the same spot. Several of these data acquisition operations have been carried out from 2015 to the present time, and are still occurring.

When analyzing such data, one has very little information to work with. There is no exhaustive list of possible events that might be taking place, and there is not any information about starting times or duration of their occurrences. If the analyst is looking for a very specific event (in terms of functional form either in time or frequency domain), it might be possible to design a (unsupervised) detection algorithm tailored to this functional form; however, the lack of information about the starting times of the events makes the detection task very computationally intensive, given the sparsity of subaquatic acoustic events [8].

A natural approach is then to, first and foremost, separate sections from the signal that are likely to contain some event (as opposed to sections containing noise only). The underwater oceanic environment is very economic in this sense: there can be periods of many minutes (even hours) where nothing but the waves (and the omnipresent sound of shrimps and barnacles clapping and clicking) can be heard. This indicates that, rather than processing the entire signal in search of an event, it would be much better to first obtain sections where we believe something is happening, and then try to figure out exactly what it is.

This problem is known in the literature as signal segmentation, and shows up in different contexts [9,10,11,12]. In the specific context of audio segmentation, [13] presents a review of the algorithms often applied to solve this problem. These are divided in three categories: the distance-based segmentation; the model-based segmentation; and hybrid techniques. The distance-based approach works directly with the audio waveform (amplitudes in time domain), and assumes nothing about the form of the segments; the model-based segmentation, on the other hand, starts with a collection of models, and works by training a classification algorithm on previously annotated data. The hybrid approach applies both distance-based and model-based approaches in a single framework.

In this paper, we propose an algorithm that can be classified as distance-based, in the sense that it does not assume a collection of models, nor does it use any pre-annotated samples. We formulate the problem in a very general form, and adopt a maximum entropy Bayesian approach to propose a solution. We argue that this theoretical framework is specially adequate for the situation of scarce information, since from maximum entropy we learn how to avoid insidious and implicit assumptions to sneak into the model, and from Bayesian inference we learn how to make the best use of whatever prior information is available.

Also, the maximum entropy Bayesian approach allows us to work from first principles, avoiding the introduction of *ad hoc* procedures. We work our way from the assumptions to the final method of solution, using little more than the basic theorems of probability theory, and making explicit each and every assumption we make about the signal we are analyzing.

The rest of the paper is organized as follows: in the next section, we introduce our main assumption and build the first part of the segmentation procedure. Section 3 completely defines our segmentation algorithm. Section 4 compares our algorithm with an alternative method found in the literature, using simulated data, and Section 5 makes the same comparison in a few samples from the actual signal obtained from the OceanPod. Section 6 concludes the paper.

## 2. Bayesian Model for Power Switch

Suppose we are given a discretely sampled signal $y\in {ℜ}^{N}$ corrupted with noise. Given a sampling frequency rate $f_{s}$, this signal corresponds to a total duration of $T=N\cdot f_{s}$ seconds. We assume that the signal is stationary with 0 mean amplitude, $E(y_{t})=0$, and finite power $Var\left(y_{t}\right)=\sigma_{0}^{2}<\infty$. We adopt the notation $y_{t}$ to indicate the *t* component of the discrete-time signal.

Now let us imagine that, at sample time $\bar{t}\in \left\{0,\ldots ,T\right\}$, some event has started. Our only assumption is that, whatever the particular nature of this event, it causes a change in the total signal power; this is a rather reasonable assumption, since for the combination of two random variables to have the same variance as one of its components, it is necessary that the variables have an exact covariance of $\sigma_{1}^{2}/2$, where $\sigma_{1}^{2}$ is the variance of either one of the variables.

If we assume only that the signal has 0 mean amplitude and finite power, the maximum entropy principle leads us to choose a Gaussian probabilistic model for *y* [3]. The maximum entropy principle indicates that, given what is known about a random variable, one must choose the probabilistic model *p* that maximizes the entropy H(p)=−∫p(x)log(p(x))dx, subject to the constraints that reflect our prior knowledge about the variable; adoption of this principle avoids the insidous inclusion of unwanted and/or unwarranted assumptions about the data in the model’s form [14].

In our case, assuming the change of variance at $t=\bar{t}$, we write the model

(1) $$y_{t}∼\left\{\begin{array}{cc} N(0,\sigma_{0}^{2}) & \;\mathrm{if}\;t\leq \bar{t}\\ N(0,\delta \sigma_{0}^{2}) & \;\mathrm{if}\;t>\bar{t} \end{array}\right.$$

The parameter δ represents the ratio between the variances of the two signal sections or segments.

Our goal is to estimate the value of $\bar{t}$, the starting time of the event. To accomplish that, we start by writing the likelihood

(2) $$L\left(\bar{t},\sigma_{0}^{2},\delta |y\right)={\left(2\pi \sigma_{0}^{2}\right)}^{-\frac{N}{2}}\delta^{-\frac{N-\bar{t}}{2}}exp\left[-\frac{\sum_{t=1}^{\bar{t}}y_{t}^{2}}{2\sigma_{0}^{2}}-\frac{\sum_{t=\bar{t}+1}^{N}y_{t}^{2}}{2\delta \sigma_{0}^{2}}\right]$$

To keep our model as general as possible, we do not assume any prior information about δ (i.e., we do not know anything about the signal to noise ratio (SNR) of the possible events). In Bayesian terms, this ammounts to adopting a non-informative (improper) uniform prior for δ. Using this prior, we are able to analytically integrate Equation (2) and obtain

(3) $$\begin{array}{cc} L(\bar{t},\sigma_{0}^{2}|y)= & \int_{0}^{\infty }L\left(\bar{t},\sigma_{0}^{2},\delta |y\right)d\delta \end{array}$$

(4) $$\begin{array}{cc} \propto & {\left[\frac{\sum_{t=\bar{t}+1}^{N}y_{t}^{2}}{2\sigma_{0}^{2}}\right]}^{-\frac{\left(N-\bar{t}-6\right)}{2}}\Gamma \left(\frac{N-\bar{t}-2}{2}\right)exp\left[-\frac{\sum_{t=1}^{\bar{t}}y_{t}^{2}}{2\sigma_{0}^{2}}\right] \end{array}$$

If the variance of either segment is known, the above equation can be used directly to obtain the posterior distribution for $\bar{t}$. If $\sigma_{0}^{2}$ is unknown, we must choose a prior distribution for it. We will not assume any particular knowledge about the variance; to keep the model invariant with relation to reparametrizations of this parameter, we adopt the Jeffreys’ prior [15,16] $\pi \left(\sigma_{0}\right)=1/\sigma_{0}$, and again integrate the above equation analytically to arrive at

(5) $$\begin{array}{cc} L(\bar{t}|y)= & \int_{0}^{\infty }L\left(\bar{t},\sigma_{0}^{2}|y\right)\pi \left(\sigma_{0}\right)d\sigma_{0}\propto {\left(\sum_{t=\bar{t}+1}^{N}y_{t}^{2}\right)}^{-\frac{\left(N-\bar{t}-6\right)}{2}}\Gamma \left(\frac{N-\bar{t}-2}{2}\right){\left(\sum_{t=1}^{\bar{t}}y_{t}^{2}\right)}^{-\frac{\left(\bar{t}+6\right)}{2}}\Gamma \left(\frac{\bar{t}+6}{2}\right) \end{array}$$

From this point, it is only necessary to pick a prior $\pi_{t}\left(t\right)$ for $\bar{t}$, multiply it by the above equation, and obtain (the kernel of) the posterior distribution for $\bar{t}$:

(6) $$\begin{array}{cc} P\left(\bar{t}|y\right)\propto \pi \left(\bar{t}\right)\cdot {\left(\sum_{t=1}^{\bar{t}}y_{t}^{2}\right)}^{-\frac{\left(\bar{t}+6\right)}{2}} & {\left(\sum_{t=\bar{t}+1}^{N}y_{t}^{2}\right)}^{-\frac{\left(N-\bar{t}-6\right)}{2}}\times \Gamma \left(\frac{\bar{t}+6}{2}\right)\Gamma \left(\frac{N-\bar{t}-2}{2}\right) \end{array}$$

This gives us a discrete distribution with support over $1<\bar{t}<N-1$; this means that it is possible to calculate exactly the normalization constant that would make (6) a proper probability distribution. If we need to estimate $\bar{t}$, on the other hand, we can optimize Equation (6) by inspection to find the MAP (Maximum a Posteriori) estimate.

**Figure 1:**
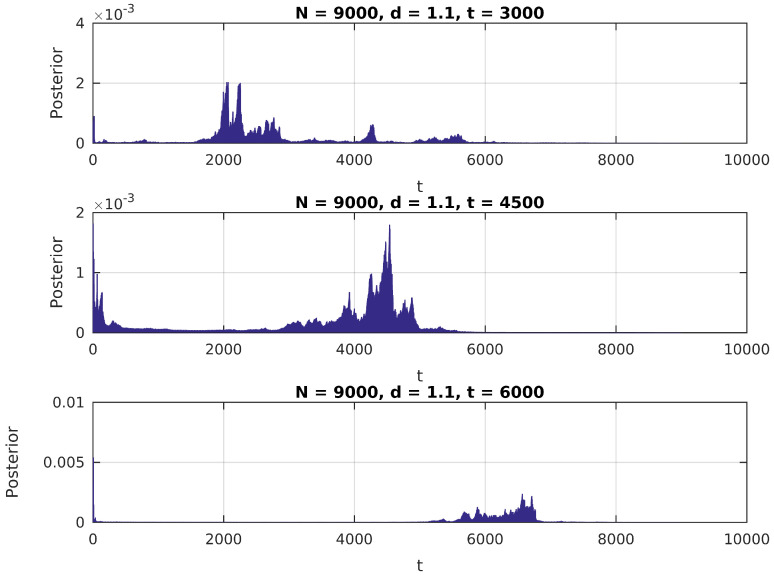
Posterior distribution for $\bar{t}$, δ=1.1.

**Figure 2:**
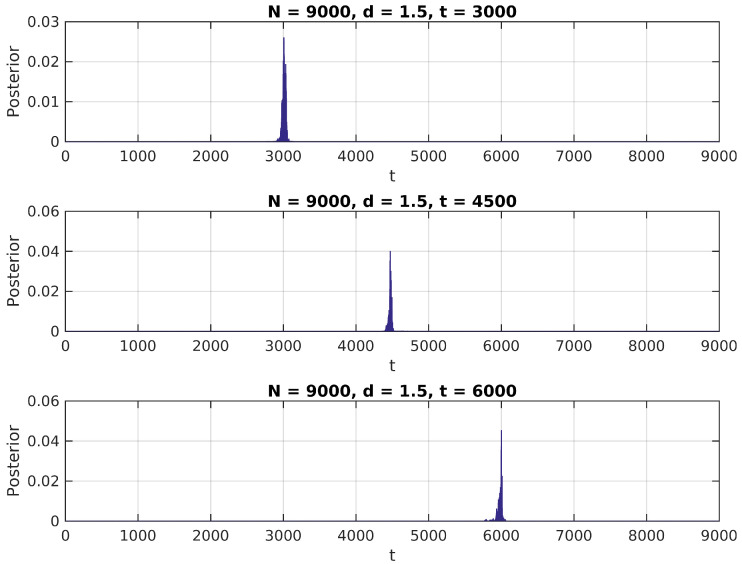
Posterior distribution for $\bar{t}$, δ=1.5.

**Figure 3:**
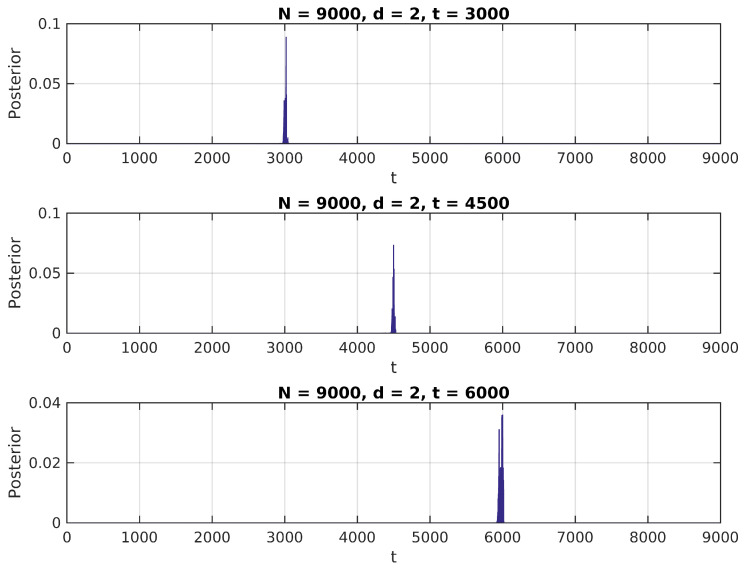
Posterior distribution for $\bar{t}$, δ=2.

In Figure 1, Figure 2 and Figure 3, we present the distribution in (6) calculated over a simulated (Gaussian) signal of size N=9000, with δ∈{1.1,1.5,2}, $\bar{t}\in \left\{N/3,N/2,2N/3\right\}$ and $\sigma_{0}=1$, and assuming a uniform prior over {2,N−2} for $\bar{t}$.

We notice in these figures a few important features of the above distribution; first, it peaks precisely around $\bar{t}$ for higher values of δ, as would be expected. Second, when it shows higher peaks far from the true value of $\bar{t}$, they are usually located near the extremes of the interval. This can be attributed to high standard error of variance estimations in small samples, and could be easily eliminated by the use of an informative prior on $\bar{t}$ (for instance, by assigning zero probability to values of *t* close to 0 or *N*).

The model for the segmentation, as defined above, works well when the signal has one cut point. In most signals, however, and certainly on the samples from the OceanPod, there will be more than one segmentation point (i.e., more than one change of signal total power).

It would be possible in theory to generalize the above model to these situations, obtaining a posterior distribution for $\left({\bar{t}}_{1},{\bar{t}}_{2},\ldots ,{\bar{t}}_{k}\right)$. However, the complexity of the discrete optimization problem involved in the MAP estimation of the cut points would grow exponentially. Also, it would be necessary to treat the new parameter *k*, the number of cutting points (or, equivalently, the number of segments in the signal), which we would like to avoid since we do not know this number in advance, and estimating it would be demanding. This new parameter is directly related to the dimensionality of the parametric space. Estimating this kind of parameter is a problem of model order selection, which, although interesting in its own right, would complicate matters excessively in the analysis of this specific problem.

**Figure 4:**
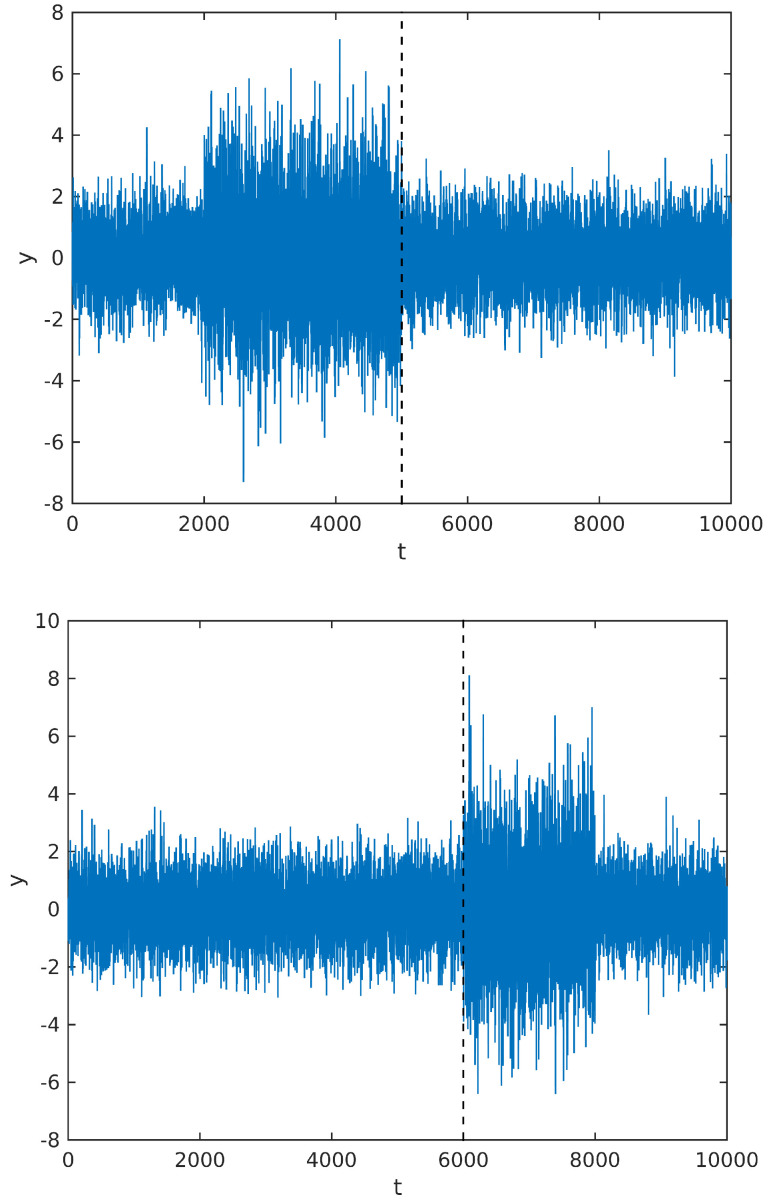
Segmentation point estimated for a signal with two power changes; see text for details.

Instead, we observe that the MAP estimate calculated from the above posterior will tend to divide the signal in regions with maximally different powers. So, whenever there is more than one change of power along the signal sample, it would still tend to estimate the cutting point at the beginning or end of one segment. To illustrate our point, we simulate a signal $y\in {ℜ}^{10,000}$ with two cutting points, in two different situations: first, we change the signal power from $\sigma_{0}=1$ to $\sigma_{1}=2$ at t=2000, and then back to $\sigma_{0}$ at t=5000; in the second figure, we change the power at t=6000 and then back to the original power at t=8000. The simulated signal, along with the cutting points estimated by maximizing the posterior distribution, are shown in Figure 4 below.

This observation suggests a recursive approach for automatic signal segmentation: we first estimate one cutting point, and divide the original signal *y* in two segments, $y_{1}$ and $y_{2}$. We then apply again the same estimation to the new signals $y_{1}$ and $y_{2}$, and so on. We repeat this procedure until some stopping criterion is met. This strategy gives rise to the sequential segmentation algorithm, which we define next.

## 3. The Sequential Segmentation Algorithm

We define the sequential segmentation algorithm as follows:

*(Sequential segmentation algorithm) Define the function **seg**($y\in {ℜ}^{N}$,$n_{min}$) as:*

1 **If** $N<n_{min}$, stop, returning the empty vector t=[];
2 **Else**
  (a) Obtain the MAP estimate $\bar{t}$;
  (b) Define $y^{1}=y_{1,\ldots ,\bar{t}}$ and $y^{2}=y_{\bar{t}+1,\ldots ,N}$;
  (c) (Stopping criterion) **If** $var\left(y^{1}\right)=var\left(y^{2}\right)$, stop, returning the empty vector t=[];
  (d) **Else** return the concatenated vector $\left[seg\left(y^{1},n_{min}\right)\;\bar{t}\;\bar{t}+seg\left(y^{2},n_{min}\right)\right]$.

Please note that the **seg** appearing in the last line refers to the function itself; our algorithm, then, is of a recursive nature.

This recursive algorithm will output an ordered vector $\tau \in {\left[1,\ldots ,N-1\right]}^{k}$ with the starting points of *k* signal segments, where the segments have been found to exhibit different powers. The condition $N\geq n_{min}$ guarantees that the algorithm stops and is well defined; the main question is how to decide if $var\left(y^{1}\right)=var\left(y^{2}\right)$, i.e., to define the stopping criterion.

As it is clear from the definition, the matter is one of testing the hypothesis of equality of variances, given two samples with mean 0. Or, using our parametrization, to test the hypothesis $H_{0}$:δ=1.

Our model is defined in the parametric space $\Theta ={ℜ}_{+}\times ℜ$, where $\theta =(\sigma_{0}^{2},\delta )$; under $H_{0}$, we have $\Theta_{0}={ℜ}_{+}$. Our hypothesis thus lies in a subspace of Θ, i.e., it is a sharp hypothesis.

The traditional statistical literature proposes a few different ways to test equality of variances, the most known being possibly the *F* test, and the likelihood ratio test. However, it is well reported that the traditional, most frequently used tests, have a few drawbacks, related in particular to the definition of the alternative hypothesis [17], or with the choice of an appropriate significance level for the decision function [18,19]. The classical Bayesian alternative would then be a Bayes factor test, which in turn would meet some difficulties with the fact that our null hypothesis defines a lower dimensional parametric space [20].

A full Bayesian procedure is available, however, that is well suited to the test of sharp hypothesis such as $H_{0}$:δ=1; this is the now well-known full Bayesian significance test (FBST) of Pereira and Stern [20]. This procedure works in the full parametric space, defining an evidence measure based on the surprise set of points having higher posterior density than the supremum posterior under $H_{0}$. The test avoids altogether the imposition of positive probabilities over null measure sets such as $\Theta_{0}:\left\{\left(\delta ,\sigma_{0}\right)\in {ℜ}^{2}:\delta =1\right\}$, and has been tested many times with good results (for a few examples, see [21,22,23,24]).

Of course, even with the application of the FBST, there remains the problem of defining a decision function over the evidence value for $H_{0}$, $ev(H_{0},y)$; as we will see, however, this decision function will become trivial when we calibrate our testing procedure with the use of appropriate priors.

Recalling the probabilistic model of our signal, given *y* and $\bar{t}$, and defining $y^{1}$ and $y^{2}$ as in the above algorithm, we write the full posterior

(7) $$P\left(d,s|y^{1},y^{2}\right)\propto \pi_{\delta }\left(d\right)\pi_{\sigma }\left(s\right){\left(2\pi s^{2}\right)}^{-\frac{n_{1}+n_{2}}{2}}d^{-\frac{n_{2}}{2}}exp\left[-\frac{\sum_{t=1}^{n_{1}}y_{1,t}^{2}}{2s^{2}}-\frac{\sum_{t=1}^{n_{2}}y_{2,t}^{2}}{2ds^{2}}\right]$$

where $n_{1}$ and $n_{2}$ are the corresponding dimensions of $y^{1}$ and $y^{2}$, $\pi_{\delta }$ is the prior for δ and $\pi_{\sigma }$ the prior for $\sigma_{0}$.

To incorporate the lack of knowledge about the base signal variance $\sigma_{0}$, and at the same time to guarantee invariance, we adopt again a Jeffreys’ prior $\pi_{\sigma }\left(s\right)=1/s$.

For δ, however, we would like to choose this time an informative prior to model our knowledge about the signal characteristics. Including an informative prior for δ will also provide the algorithm with a calibration parameter, that will allow the method to be adapted to different circumstances (different lengths for the signal sample, different SNR characteristics, etc.).

To define this prior, we consider what we do know about δ. We reason in the following terms: suppose we are to pick at random two contiguous sections of our signal, with sizes $n_{1}$ and $n_{2}$; suppose that these sections have $s_{1}$ and $s_{2}$ as their respective powers, as estimated from the data. We then define $\delta =\frac{s_{2}}{s_{1}}$.

Now, unless we happen to pick by chance two segments that include a true change of power (in our terms, the beginning or end of an event), we expect δ to be very close to 1. This belief would be as strong as our perceived probability of finding an event at random in our signal. As we have mentioned before, the ocean’s subacquatic soundscape is a rather minimalist environment, with long periods of very low or no activity. So we believe that, in our thought experiment above, we are very likely to pick sections with δ very close to 1.

However, if we happen to pick a segment with an event, then we can expect to find δ>>1, since most events in our signal have large SNR values (for instance, the already mentioned boat engines, running at a small distance from the hydrophone). It is then reasonable to believe that, even though δ is likely close to 1, it can sometimes differ significantly and assume high values, close to δ=2 or even higher.

All of these considerations lead us to pick a prior distribution on δ that is (a) centered around 1; (b) high-peaked around this same value; but (c) with larger tails than the Gaussian. Further on, to keep matters as simple as possible, we would like our prior to have few parameters (since we will use these parameters for the calibration of our algorithm). Combining all of these objectives, we propose a Laplace prior for δ:

(8) $$\pi_{\delta }\left(d\right)=\frac{1}{\beta }e^{-\frac{\left|d-1\right|}{\beta }}$$

In practical terms, this prior will tend to favor $H_{0}$:δ=1, inversely with the value of β. This value can be used as a calibration parameter for the detection algorithm. Also, picking a sufficiently low value for β will guarantee a minimum prior probability for the meaningless situation δ≤0.

Again, we must stress that in working with acoustic signals with a sampling rate as high as 11 kHz, we will be dealing with large sample sizes; typically, we will define a smallest detectable event as a segment with a duration of around 1 s, which means that we will be comparing the variances of samples with sizes *N* = 11,025 each. On the other extreme, the algorithm will start with a signal of duration in the order of minutes (the files obtained from the hydrophone are configured as 15 min long by default), which translates to sample sizes in the order of millions. Our numerical tests indicate that the value of β must be set correspondingly; our best results used $\beta \propto 10^{-5}$, as we will see in the results section.

With the model completely specified, the evidence value against $H_{0}$ is calculated as

(9) $$ev\left(H_{0},y\right)=\int_{T(y)}P\left(\sigma_{0},\delta |y\right)d\sigma_{0}d\delta$$

where

(10) $$T\left(y\right)=\{\left(\sigma_{0},\delta \right)\in \Theta :P\left(\sigma_{0},\delta |y\right)>sup_{\Theta_{0}}P\left(\sigma_{0},\delta |y\right)\}$$

The value of the integral of the posterior over the surprise set is high whenever the manifold defining $\Theta_{0}$ traverses regions of low posterior probability. In this case, the evidence against $H_{0}$, given by the above Equation (9) will also be high.

It is noteworthy that, when defining the posterior for the cutting point $\bar{t}$, we chose priors for all the other parameters in order to analytically integrate them out. The intention behind this decision was to keep this step of the algorithm as simple as possible, since MAP estimation in this case is a discrete optimization problem which we solve by brute force. Now, however, when calculating the evidence for the hypothesis δ=1, we want to work on the full parametric space, without explicitly marginalizing any parameter. This is the standard procedure when working with the FBST [20].

To calculate the above integral, then, we adopt a numerical procedure: we apply a block Metropolis–Hastings algorithm, with a sample size of 10,000 points after burning another 10,000 points, and using exponential distributions as candidates for both σ and δ.

The stopping criterion for the algorithm is finally defined by setting a minimal evidence for $H_{0}$, $\alpha_{min}$; the algorithm will keep segmenting the signal as long as the evidence for $H_{0}:\delta =1$ is lower than this threshold value:

*(Stopping criterion) Given $y^{1}$, $y^{2}$ and $\alpha_{min}$:*

1. Obtain $s_{0}=sup_{\Theta_{0}}P\left(\sigma_{0},\delta |y\right)$;
2. Obtain the evidence favoring $H_{0}$ : $ev\left(H_{0},y\right)=1-\int_{T(y)}P\left(\sigma_{0},\delta |y\right)d\sigma_{0}d\delta$;
3. **If** $ev\left(H_{0},y\right)<\alpha_{min}$, return 1 ($var\left(y^{1}\right)\neq var\left(y^{2}\right)$); **else** return 0.

**Figure 5:**
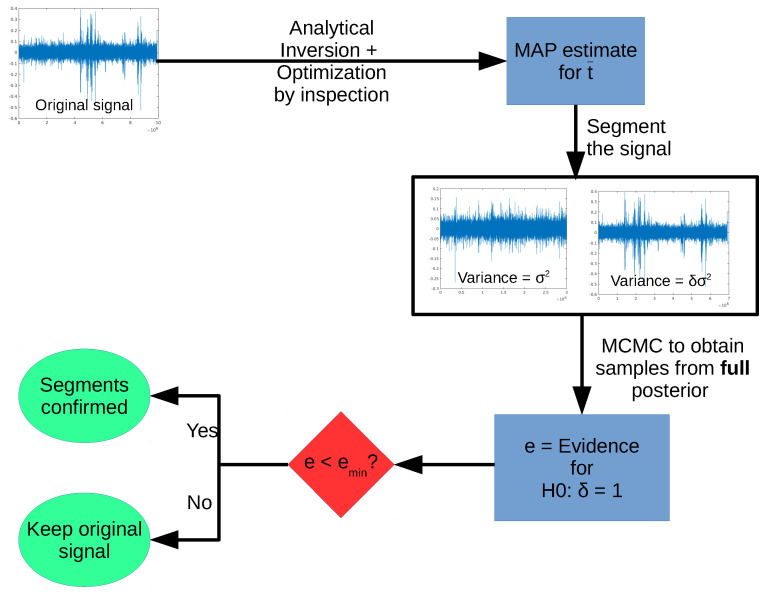
One step of the sequential segmentation algorithm.

One step of the full algorithm is shown schematically in the diagram in Figure 5. Starting with a given signal, we first obtain the MAP estimate for $\bar{t}$, optimizing by inspection the posterior for the change point *t*. After estimating $\bar{t}$, we generate two segments, and use the FBST (via Markov Chain Monte Carlo (MCMC) sampling of the full posterior) to test the hypothesis $H_{0}$:δ=1.

## 4. Results: Simulated Signal

To test the performance of our algorithm, we first apply it to simulated data. We simulate $y\in {ℜ}^{20,000}$, where $y_{t}∼N\left(0,\sigma_{i}^{2}\right)$, and we define five segments in the signal, given by the following definition for the variance $\sigma_{i}^{2}$:

(11) $$\sigma_{i}^{2}=\left\{\begin{array}{cc} 1 & \;\mathrm{if}\;i\leq 5000\\ 1.1 & \;\mathrm{if}\;5000<i\leq 10,000\\ 1 & \;\mathrm{if}\;10,000<i\leq 12,000\\ 1.5 & \;\mathrm{if}\;12,000<i\leq 15,000\\ 1 & \;\mathrm{if}\;i>15,000 \end{array}\right.$$

As a baseline method to use for comparison, we adopt the peak detection algorithm of Palshikar [25]. This algorithm defines peaks in the signal by using local and global properties. The local properties are based on functions calculated over windows of width *k*, centered around each coordinate of the signal. Each function reflects a different characterization of what actually constitutes a peak. The global properties arise when each peak is compared to the average amplitude of all peaks, and a thresholding is applied based on their standard deviation.

Palshikar’s algorithm takes then two parameters: *h*, the number of standard deviations to use as threshold, and *k*, the length of the window. Also, the algorithm can use many different functions to define a peak locally; we run Palshikar’s algorithm using his three *S* functions. The first function, $S_{1}$ calculates for each point $y_{t}$ of the signal the maximum (signed) difference between $y_{t}$ and its left neighbors (points with indexes in the range *j*:j<t&|t−j|<k), the maximum (signed) difference between $y_{t}$ and its right neighbors ($\{y_{j}$:j>t&|t−j|<k}), and takes the average between these two maxima. The second function, $S_{2}$, takes the mean (signed) difference between $y_{t}$ and its left neighbors, the mean (signed) difference between $y_{t}$ and its right neighbors, and takes the average between the two means. The third function, $S_{3}$, takes the signed difference between $y_{t}$ and the average of its left neighbors, the signed difference between $y_{t}$ and the average of its right neighbors, and takes the mean. For details about the algorithm and a review on peak detection methods, we refer the interested reader to Pashilkar’s paper [25].

In Table 1, we compare the results of our method with Palshikar’s algorithm, using each of the three *S* functions and eight combinations of values for *h* and *k*. For the sequential segmentation algorithm, we set β∈{1,0.01} and α∈{0.01,0.1}; lower values of β imply higher prior weight to the hypothesis δ=1, while lower values of α imply earlier stop for the algorithm (i.e., we require higher evidence against δ=1 to keep segmenting the signal). In the table, we labelled our algorithm as SeqSeg.

The experimental results show that our algorithm is much more robust than the peak detection algorithm of Palshikar. Every combination of parameters we used resulted in the same output, correctly segmenting the signal into five segments, the first starting around i=5000, and the last around i=15,000. The peak detection algorithm is much more sensitive to the parameters choice. The best result for the peak detection algorithm was using $S_{1}$, with h=3 and k=500. Also, it is clear that Palshikar’s algorithm performed much better when the segment’s SNR was higher (i.e., it worked better identifying the last cutpoint, rather than the first); even though our algorithm will also perform better with higher SNR, the value of δ=1.5 is sufficient for the algorithm to correctly capture the first segment.

On the other hand, our algorithm is much slower than Palshikar’s. This is due to the fact that our method involves one brute-force, integer optimization step, and one MCMC step. Nevertheless, this paper was written using a pilot version of our algorithm, written in MATLAB^®^ with little concern for computational performance. In particular, no parallelization strategy was adopted, and both steps are strongly parallelizable. With this issue in mind, we are working on a new version of the algorithm implemented in Python and parallelized using the multiprocessing library; we expect this new version to sensibly improve the computational performance of our algorithm.

There is, however, one quick way to improve the performance of the SeqSeg method, in particular of the brute force optimization step. We can set a time resolution for the algorithm, and instead of calculating the posterior for each and every t=1,…,N, we can evaluate it at equally spaced points t=r,2r,…,k·r. The resolution *r* can be set to any value which is less than the length of a typical event, and the number of function evaluations in the optimization step decreases linearly with *r*.

## 5. Results: OceanPod Samples

Our main goal when developing the segmentation algorithm was to annotate samples from the OceanPod, in search of segments that are likely to capture any significant event. These segments can then be analyzed on their own, to characterize the events and build an annotated database to be fed to a classification algorithm.

It is important to once again stress that we do not have previous knowledge about the events taking place in the OceanPod signal; this means that it is difficult to define a precise performance measure to any segmentation or annotation algorithm applied to these data. What we propose to do is to select a few samples, with distinctive characteristics, and manually count the number of segments we expect in each sample (by inspecting the spectrogram, where the events are more easily spotted). Then, we can compare the results of the segmentation algorithm to this number.

For that, we select three samples from the OceanPod signal, each of them with a total length of 15 min (the default filesize for the OceanPod). The first is a recording from 30 January 2015, Saturday, from 02:02:56 to 02:17:56. During this period, there is no perceivable activity beyond background noise (concentrated around 5 kHz). When applying any segmentation method to this sample, we would then expect no segments to be found. The second is a recording from 2 February 2015, Monday, from 07:50:49 to 08:04:49. In this sample, we find a long duration event, starting at time 0 and lasting for approximately 10 min. By listening to the sample, we identify the sound of a large sized vessel, passing by at a long distance and with low speed. The segmentation algorithm applied to this sample should detect one or two change points for the signal’s power, ideally forming a segment starting at i=0 and ending around *i* = 6,615,000, i.e., 10 min into the signal (recall that the sampling rate is $f_{s}=11.025$ Hz).

The third sample is from 8 February 2015, Sunday, from 11:26:39 to 11:41:39. During this 15 min, there are many events taking place; listening to this sample, we identify the engine of smaller vessels, being turned on and off and near the hydrophone spot. In this sample, we expect the segmentation algorithm to detect many events; by visually inspecting this sample, we identify 32 distinct segments.

**Figure 6:**
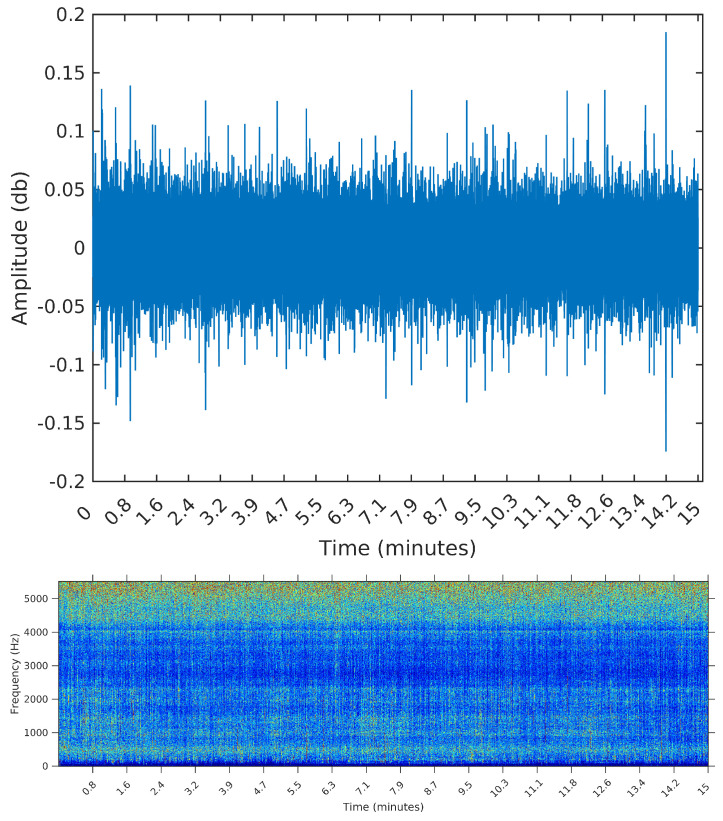
Waveform and spectrogram of the first sample: noise only.

**Figure 7:**
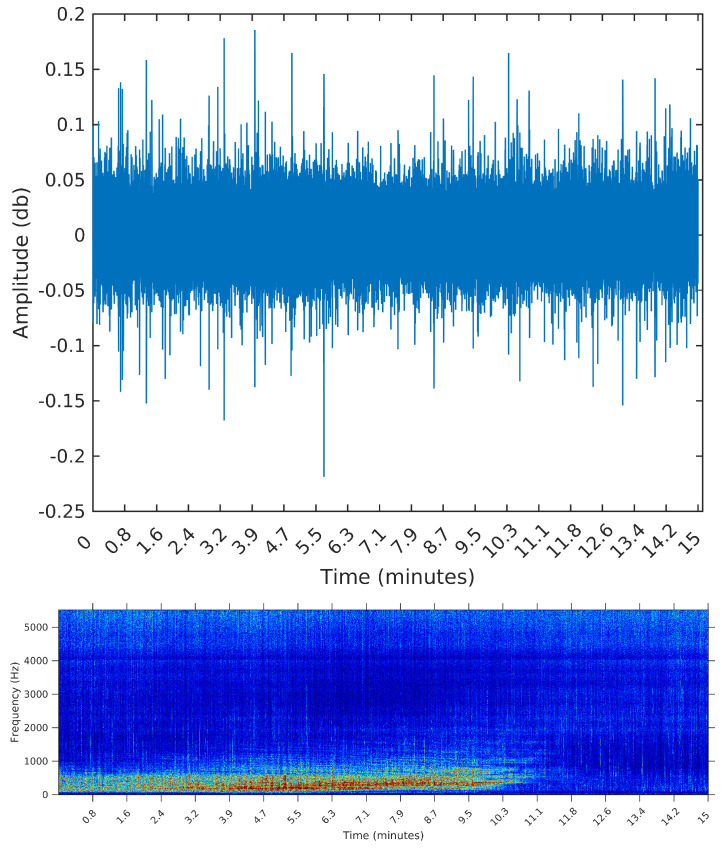
Waveform and spectrogram of the second sample: one long duration event.

**Figure 8:**
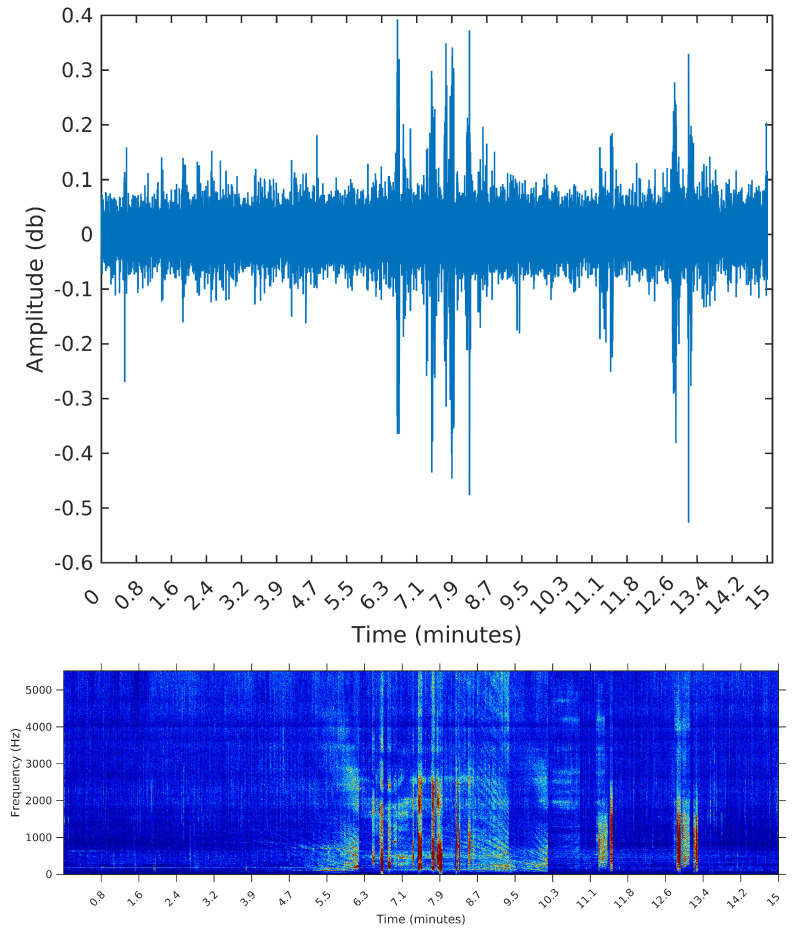
Waveform and spectrogram of the third sample: many short events.

We opted to show in Figure 6, Figure 7 and Figure 8 the spectrograms obtained for each of the three samples, even though the segmentation algorithm does not use any feature in the frequency domain. The reason for using the spectrograms, in addition to the raw waveforms, is that we found it much easier to spot the events, specially in the third sample, when plotting on the frequency domain. An event, or segment, in these pictures, is a “heater” (red instead of blue) region on the spectrogram. We are interested in obtaining the time (horizontal) coordinates of each of these events, regardless of their spectrum (vertical coordinate).

We apply our sequential segmentation algorithm to each of these samples; we take $\beta \in \{3\times 10^{-5},1\times 10^{-5}\}$ and fix α=0.01. Our experiments show that higher values for β result in an excess of segments. For comparison, we also apply Palshikar’s algorithm to the same samples, using the $S_{1}$ function, with k=11.025 fixed and h∈{3,5}.

Because both our algorithm and Palshikar’s involve calculation of some function (the posterior distribution for $\bar{t}$, in our case, and the function $S_{i}$ for Palshikar’s) for each coordinate $y_{t}$ of the signal vector, when analyzing samples as long as 15 min, with a sampling frequency of 11.025 Hz, the performance of both algorithms became impractical (more than 10 min to process each sample). To deal with this situation, we adopt the strategy of fixing a time resolution r=11.025 to our algorithm, and a time resolution of r=10 to Palshikar’s. This means that we will calculate the posterior only for $y_{j\cdot 11.025},j=1,\ldots ,floor\left(N/11.025\right)$, and the *S* function for $y_{j\cdot 10},j=1,\ldots ,floor\left(N/10\right)$.

We have experimented with using r=11.025 for both methods. This accelerated the peak detection algorithm to running times of little more than 1 s. However, the nature of the *S* function (being based on the maxima or averages of blocks with size *k*), caused Palshikar’s algorithm to miss many segments’ endpoints. Our experiments showed that picking r=10 was a good compromise between performance and sensibility.

Table 2 summarizes the results of both algorithms applied to each of the three samples. We present the elapsed time, in seconds, the final number of segments, and the start time in minutes of the first and last segments.

The sample from 30 January 2015 is the sample containing only noise. We then expect the algorithms to output 0 segments. This was the case only for the SeqSeg algorithm when $\beta =3e^{-5}$. Palshikar’s algorithm returned many false positives for this sample, regardless of the parameter value.

The sample from 2 February 2015 is the sample containing a long event, starting at the very beginning of the file (0 min) and ending around 10 min. Again, the SeqSeg algorithm found a much lower number of segments and correctly identified the end of the main event at around 10 min.

Finally, the sample from 8 February 2015 contains 32 segments evident to the eye. The SeqSeg algorithm came near to this number when $\beta =1e^{-5}$. It is noteworthy that, regardless of the value of β, the first and last segments found by SeqSeg had precisely the same starting points (this will always be the case, since calculation of the change points is completely deterministic). Palshikar’s algorithm came near to the true value of segments when h=5, and was less assertive in terms of the starting times of the first and last segments.

**Figure 9:**
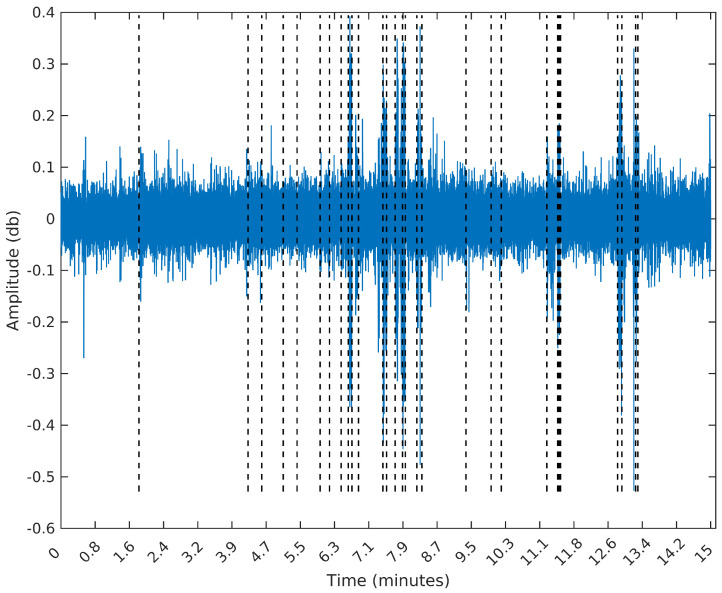

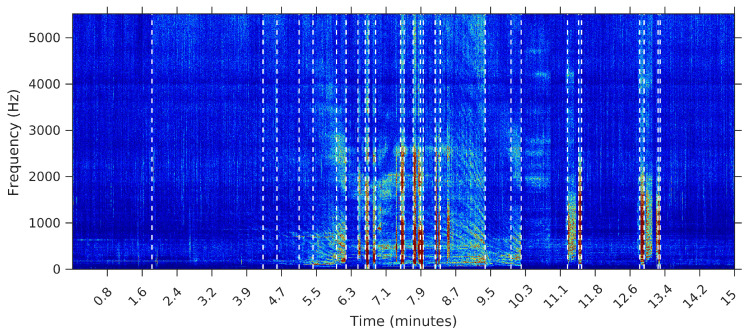
Segmentation using the SeqSeg algorithm with $\beta =3e^{-5}$.

**Figure 10:**
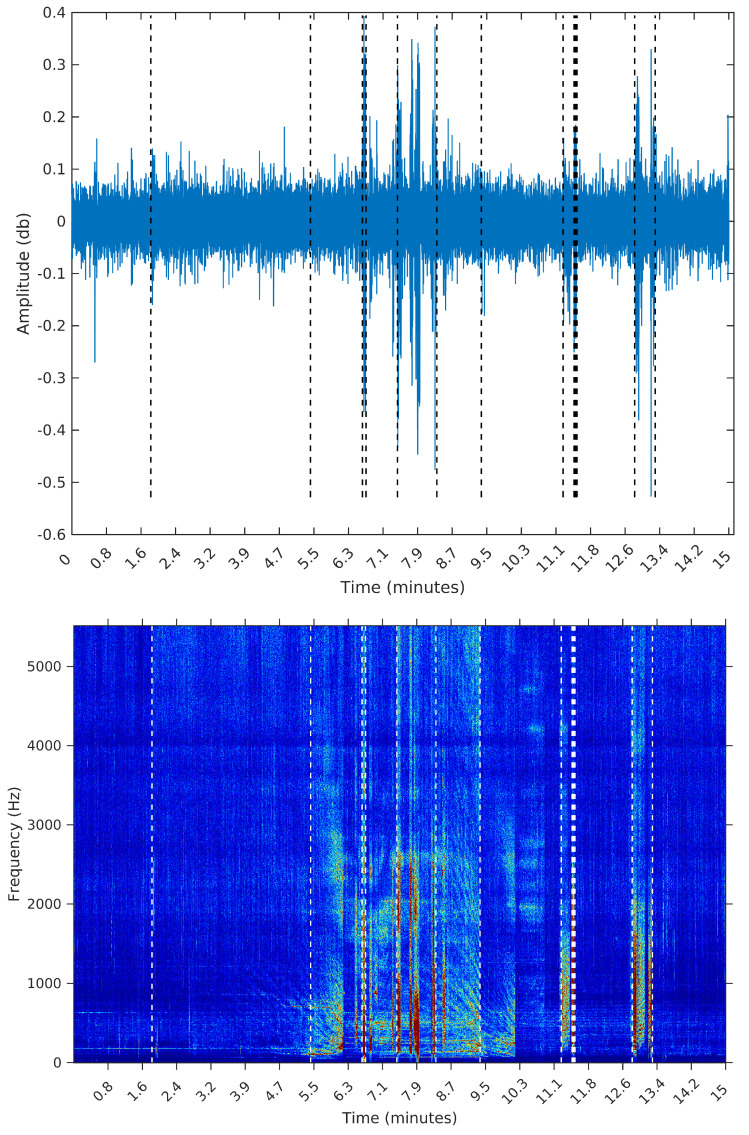
Segmentation using the SeqSeg algorithm with $\beta =1e^{-5}$.

**Figure 11:**
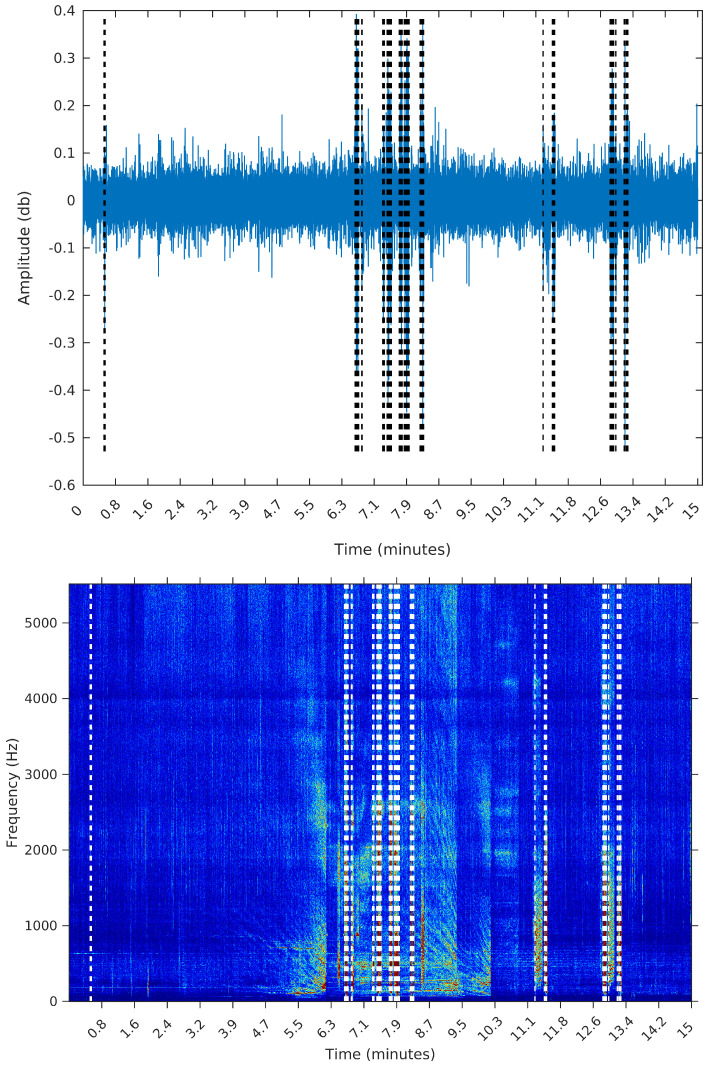
Segmentation using Palshikar’s algorithm with h=3.

**Figure 12:**
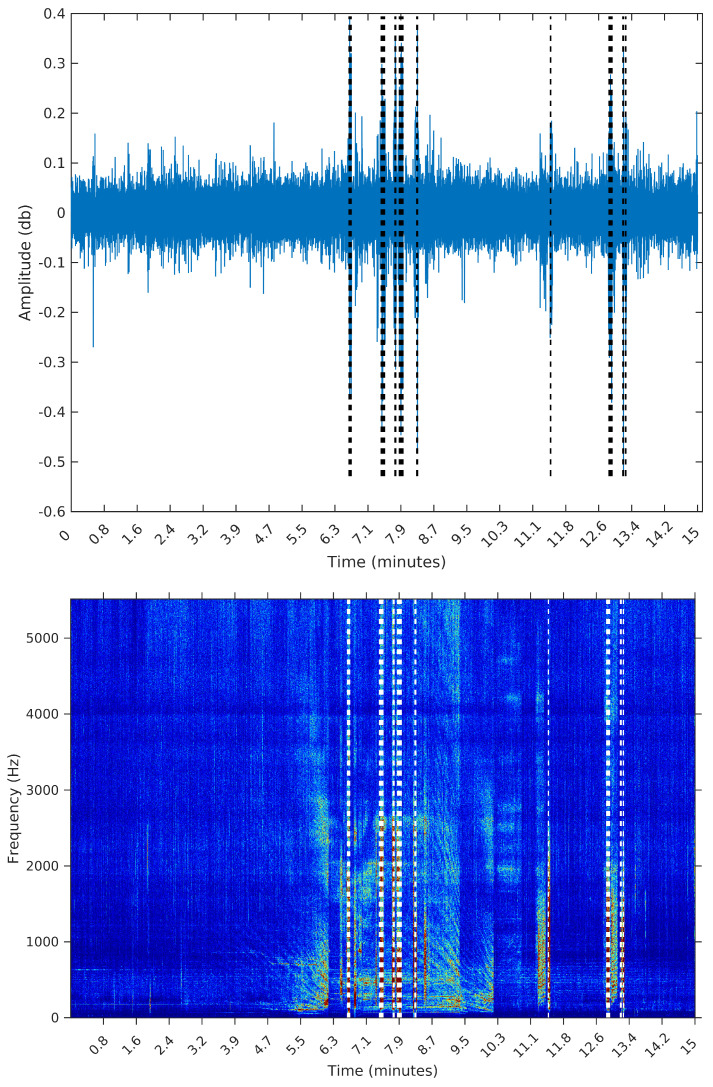
Segmentation using Palshikar’s algorithm with h=5.

To make the comparison easier between the segments obtained by the sequential segmentation algorithm and Palshikar’s algorithm in the sample from 8 February 2015, we plot in Figure 9, Figure 10, Figure 11 and Figure 12 below the waveform and spectrogram of this sample with the segments found by each algorithm represented by dashed black (on the waveform) or white (on the spectrogram) lines.

It is clear that the segments defined by the SeqSeg algorithm are much closer to what we can visually identify as meaningful events. The peak detection algorithm tends to oversegment the eventful sections, and ignore some points where there is a clear change in the signal’s power.

Finally, when applying both algorithms to real data, the computational performance of the two methods was not very different. We recall, however, that we imposed a resolution limitation to the SeqSeg algorithm. Nevertheless, even with this limitation, its results are clearly superior to the traditional peak detection strategy.

## 6. Conclusions

Our goal in this paper was to define a signal segmentation algorithm that could be as general as possible. We wanted it to be general, especially because we do not know in advance exactly what kinds of patterns (events) there might be in the data. In this sense, this algorithm can be described as an unsupervised learning method, and could be applied to the analysis of any signal or time series where the assumptions hold.

When compared to a standard peak detection algorithm, our method has shown to be much more robust and also more precise in detecting the number of events, and also their boundaries, both on simulated and real data. In practice, there is only one parameter to be calibrated (the prior’s β). The value of this parameter can be set by a technician with no mathematical training, by choosing an adequate sample to use for tuning the algorithm’s behavior.

Also, our method was built based on a simple assumption (that a segment involves a change in the signal’s energy), and based entirely on methods of Bayesian inference. There are no adhockeries involved, the closest to an *ad hoc* procedure being the choice of the prior distributions. However, we have argued for the choice of each of the priors, and we believe that all of them honestly reflect and incorporate the prior knowledge that we have about the signal and the segments.

Most (if not all) automatic segmentation algorithms currently known to the literature, on the contrary, rely on more or less arbitrary definitions and parameters (such as the form of the *S* functions or the parameter *h*). We believe that, for this reason, the calibration of such algorithms is much more difficult than in our method, and our tests show that this is indeed the case.

On the other hand, the computational performance of our method is far from ideal. We acknowledge that and are currently working on a new and parallelized version of the algorithm, that we hope will perform significantly better.

There are also other ways to improve the performance of our algorithm, the simplest way being to use smaller sample sizes for the MCMC algorithm. Experiments show that the main results are unaltered if we use samples of size 5000 instead of 10,000. Also, there are other methods to obtain samples from a posterior distribution, in particular the Approximate Bayes Computation techniques [26,27,28,29], that might help reduce the computation time, especially in the FBST step. We are currently investigating these possibilities.

We believe, however, and since our algorithm is aimed at being used as a preprocessing tool, that, based on the quality of our results, the current performance is tolerable.

## Figures and Tables

**Table 1:** Test results; see text for details.

Algorithm	Parameters	Elapsed Time (s)	# of Segments	First Cutting Point	Last Cutting Point
SeqSeg	β=1,α=0.01	9.8912	5	4990	15,001
SeqSeg	β=0.01,α=0.01	11.8567	5	4990	15,001
SeqSeg	β=1,α=0.1	12.7605	5	4990	15,001
SeqSeg	β=0.01,α=0.1	11.5746	5	4990	15,001
Palshikar $S_{1}$	h=3,k=100	0.3116	31	4778	14,852
Palshikar $S_{2}$	h=3,k=100	0.3303	13	5039	14,470
Palshikar $S_{3}$	h=3,k=100	0.3443	9	1608	14,249
Palshikar $S_{1}$	h=3,k=500	0.4264	5	5039	14,179
Palshikar $S_{2}$	h=3,k=500	1.4768	55	1608	19,892
Palshikar $S_{3}$	h=3,k=500	1.3090	25	964	19,892
Palshikar $S_{1}$	h=3,k=1000	1.0846	16	964	19,892
Palshikar $S_{2}$	h=3,k=1000	1.2406	10	964	19,892
Palshikar $S_{3}$	h=3,k=1000	1.4050	55	1608	19,892
Palshikar $S_{1}$	h=3,k=2000	0.9451	25	964	19,892
Palshikar $S_{2}$	h=3,k=2000	0.9889	16	964	19,892
Palshikar $S_{3}$	h=3,k=2000	1.1212	10	964	19,892
Palshikar $S_{1}$	h=4,k=100	0.1718	5	13,171	14,311
Palshikar $S_{2}$	h=4,k=100	0.2303	3	13,171	14,311
Palshikar $S_{3}$	h=4,k=100	0.3023	4	12,606	14,727
Palshikar $S_{1}$	h=4,k=500	0.4439	3	12,606	14,727
Palshikar $S_{2}$	h=4,k=500	0.8924	16	1608	14,923
Palshikar $S_{3}$	h=4,k=500	0.9922	10	1608	14,923
Palshikar $S_{1}$	h=4,k=1000	1.0793	9	1608	14,727
Palshikar $S_{2}$	h=4,k=1000	1.2486	6	1608	14,311
Palshikar $S_{3}$	h=4,k=1000	0.8439	16	1608	14,923
Palshikar $S_{1}$	h=4,k=2000	0.9167	10	1608	14,923
Palshikar $S_{2}$	h=4,k=2000	1.0030	9	1608	14,727
Palshikar $S_{3}$	h=4,k=2000	1.1367	6	1608	14,311

**Table 2:** Results in real signal samples; see text for details.

Algorithm	Sample	Elapsed Time (s)	Segments	First Segment (min)	Last Segment (min)
SeqSeg, $\beta =1e^{-5}$	8 February 2015	245.41	28	1.80	13.32
SeqSeg, $\beta =3e^{-5}$	8 February 2015	174.13	13	1.80	13.32
Palshikar h=3	8 February 2015	113.33	56	0.51	13.30
Palshikar h=5	8 February 2015	112.23	29	6.65	13.28
SeqSeg, $\beta =1e^{-5}$	30 January 2015	149.61	3	2.02	10.63
SeqSeg, $\beta =3e^{-5}$	30 January 2015	44.83	0	-	-
Palshikar h=3	30 January 2015	88.27	130	0.00	14.91
Palshikar h=5	30 January 2015	87.30	27	0.30	14.21
SeqSeg, $\beta =1e^{-5}$	2 February 2015	101.45	7	1.77	10.90
SeqSeg, $\beta =3e^{-5}$	2 February 2015	94.24	3	3.75	10.32
Palshikar h=3	2 February 2015	89.07	114	0.00	14.83
Palshikar h=5	2 February 2015	91.15	22	0.69	14.20
